# Crystal structure of di­methyl­ammonium hydrogen oxalate hemi(oxalic acid)

**DOI:** 10.1107/S2056989015005964

**Published:** 2015-04-11

**Authors:** Waly Diallo, Ndongo Gueye, Aurélien Crochet, Laurent Plasseraud, Hélène Cattey

**Affiliations:** a Laboratoire de Chimie Minérale et Analytique (LACHIMIA), Département de Chimie, Faculté des Sciences et Techniques, Université Cheikh Anta Diop, Dakar, Senegal; b Department of Chemistry, University of Fribourg, Chemin des Musée 9, CH-1700 Fribourg, Switzerland; c ICMUB UMR 6302, Université de Bourgogne, Faculté des Sciences, 9 avenue Alain Savary, 21000 Dijon, France

**Keywords:** crystal structure, organic salt, hydrogen bonding, hydrogenoxalate, dialkyammonium, oxalic acid.

## Abstract

Single crystals of the title salt, Me_2_NH_2_^+^·HC_2_O_4_^−^·0.5H_2_C_2_O_4_, were isolated as a side product from the reaction involving Me_2_NH, H_2_C_2_O_4_ and Sn(*n*-Bu)_3_Cl in a 1:2 ratio in methanol or by the reaction of the (Me_2_NH_2_)_2_C_2_O_4_ salt and Sn(CH_3_)_3_Cl in a 2:1 ratio in ethanol. The asymmetric unit comprises a di­methyl­ammonium cation (Me_2_NH_2_^+^), an hydrogenoxalate anion (HC_2_O_4_^−^), and half a mol­ecule of oxalic acid (H_2_C_2_O_4_) situated about an inversion center. From a supra­molecular point of view, the three components inter­act together *via* hydrogen bonding. The Me_2_NH_2_^+^ cations and the HC_2_O_4_^−^ anions are in close proximity through bifurcated N—H⋯(O,O) hydrogen bonds, while the HC_2_O_4_^−^ anions are organized into infinite chains *via* O—H⋯O hydrogen bonds, propagating along the *a*-axis direction. In addition, the oxalic acid (H_2_C_2_O_4_) mol­ecules play the role of connectors between these chains. Both the carbonyl and hydroxyl groups of each diacid are involved in four inter­molecular inter­actions with two Me_2_NH_2_^+^ and two HC_2_O_4_^−^ ions of four distinct polymeric chains, *via* two N—H⋯O and two O—H⋯O hydrogen bonds, respectively. The resulting mol­ecular assembly can be viewed as a two-dimensional bilayer-like arrangement lying parallel to (010), and reinforced by a C—H⋯O hydrogen bond.

## Abstract 2

The title salt consists of a di­methyl­ammonium cation (Me_2_NH_2_^+^), an hydrogenoxalate anion (HC_2_O_4_^−^), and half a mol­ecule of oxalic acid (H_2_C_2_O_4_) situated about an inversion center. They are linked together through inter­molecular hydrogen bonds, forming a two-dimensional bilayer-like self-assembly.

## Chemical context   

Within the scope of our research on the crystal structure determination of new organotin compounds containing dialkyammonium, we recently reported the structures of bis­(di­methyl­ammonium) tetra­chlorido­dimethyl­stannate(IV) [Diop *et al.*, 2011 ▸] and di­methyl­ammonium di­chlorido­tri­phenyl­stannate(IV) [Sow *et al.*, 2012 ▸]. Continuing our quest in this field, we report herein on the crystal structure of the title salt, Me_2_NH_2_^+^·HC_2_O_4_^−^·0.5H_2_C_2_O_4_, isolated from two distinct reaction pathways, *viz*. mixing Me_2_NH, H_2_C_2_O_4_ and SnBu_3_Cl in methanol or the reaction of the (Me_2_NH_2_)_2_C_2_O_4_ salt and Sn(CH_3_)_3_Cl in ethanol.
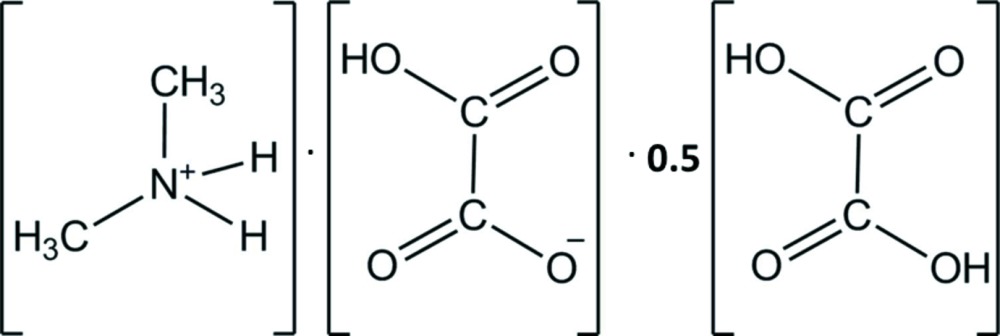


The title salt constitutes a new example of di­alkyl­ammonium hydrogenoxalates and thus supplements the number of crystal structures resolved to date for this type of salt (Birnbaum, 1972 ▸; Thomas & Pramatus, 1975 ▸; Thomas, 1977 ▸; Gündisch *et al.*, 2001 ▸; Warden *et al.*, 2005 ▸). In addition, and because of their capacity to easily develop hydrogen-bonding networks, carb­oxy­lic acids and their deriv­atives are of great inter­est in the field of crystal engineering, leading to a large diversity of supra­molecular topologies (Ivasenko & Perepichka, 2011 ▸).

## Structural comments   

**Figure 1:**
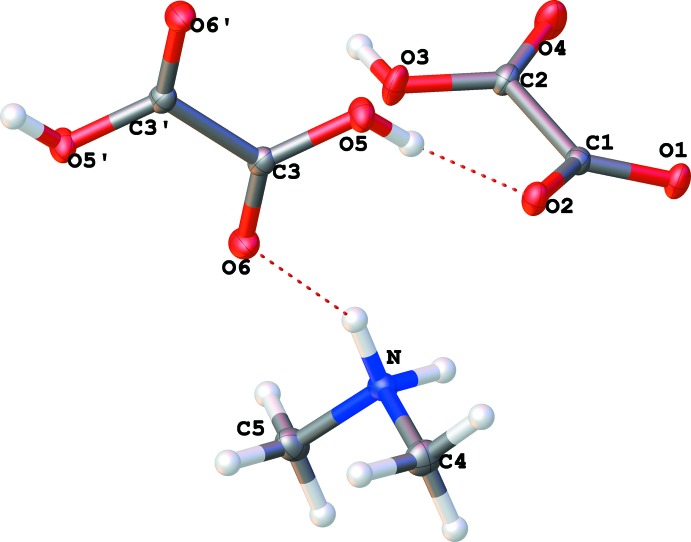
A view of the mol­ecular structure of the title salt, with the atom labelling. Displacement ellipsoids are drawn at the 30% probability level.

**Figure 2:**
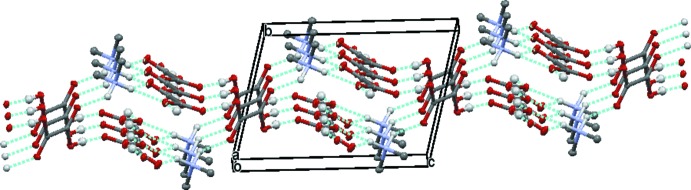
Crystal packing of the title salt, viewed along the *a* axis, showing the two-dimensional bilayer-like arrangement formed through N—H⋯O and O—H⋯O hydrogen bonds (dashed lines; details are given in Table 1 ▸). H atoms not involved in hydrogen bonding have been omitted for clarity.

In the asymmetric unit of the title salt there are three components: one di­methyl­ammonium cation (Me_2_NH_2_^+^), one hydrogenoxalate anion (HC_2_O_4_^−^), and half a mol­ecule of oxalic acid (H_2_C_2_O_4_) which possess inversion symmetry (Fig. 1 ▸). All three entities are linked by inter­molecular inter­actions (Table 1 ▸ and Fig. 2 ▸). The Me_2_NH_2_^+^ cation is in close proximity with the HC_2_O_4_^−^ anion through bifurcated N—H⋯(O,O) hydrogen bonds [N1—H1*A*⋯O1 = 2.854 (1) Å and N1—H1*A*⋯O4 = 2.964 (1) Å]. The lengths of the N–C bonds [N1—C4 = 1.4822 (12) and N1—C5 = 1.4842 (12) Å] are nearly identical of those reported previously for Me_2_NH_2_^+^·HC_2_O_4_^−^ (Thomas, 1977 ▸). The Me_2_NH_2_^+^ cation is also involved in hydrogen bonding with one of the two carbonyl groups of the oxalic acid mol­ecule [N1—H1*B*⋯O6 = 2.846 (1) Å]. The HC_2_O_4_^−^ hydrogenoxalate anions form a one-dimensional chain along the *a*-axis direction *via* the formation of O—H⋯O hydrogen bonds [O3—H3⋯O1 = 2.564 (1) Å]. Furthermore, the HC_2_O_4_^−^ anion is also involved in hydrogen bonding with one of the two hydroxyl groups of the oxalic acid mol­ecule [O5—H5⋯O2 = 2.565 (1) Å].

## Supra­molecular features   

**Figure 3:**
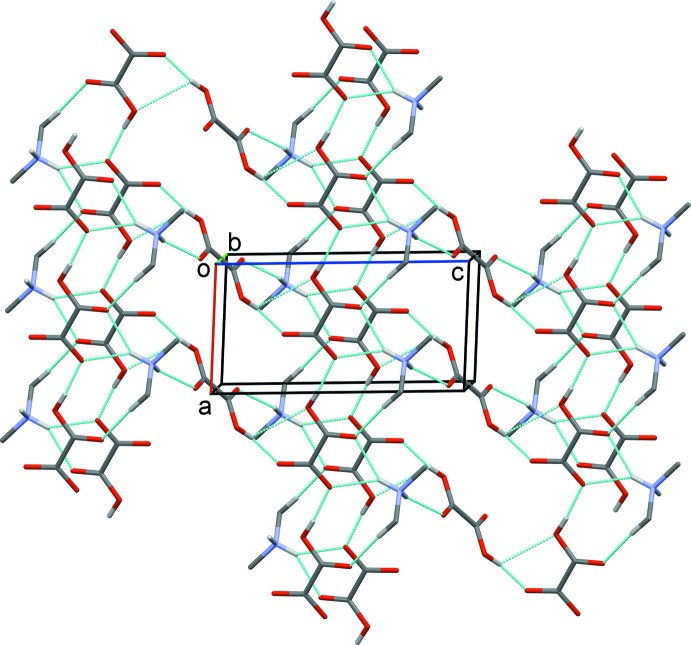
Crystal packing of the title salt viewed along the *b* axis. The hydrogen bonds are shown as dashed lines (see Table 1 ▸ for details) and H atoms not involved in hydrogen bonding have been omitted for clarity.

From a supra­molecular point of view, the combination of these inter­molecular inter­actions leads to the formation of a mol­ecular assembly which can be described as a two-dimensional bilayer-like arrangement, parallel to (010), consisting of anti­parallel infinite chains of Me_2_NH_2_^+^·HC_2_O_4_^−^ (Table 1 ▸ and Fig. 3 ▸), with an inter-chain distance of *ca* 3.0 Å. The oxalic acid mol­ecules are organized in a parallel offset fashion, and act as hydrogen-bond connectors between the chains, involving both the carbonyl and hydroxyl groups (Table 1 ▸ and Figs. 2 ▸ and 3 ▸).

## Database survey   

The crystal structure of Me_2_NH_2_^+^·HC_2_O_4_^−^, first reported by Thomas & Pramatus (1975 ▸) and then completed in 1977 (Thomas, 1977 ▸), shows a supra­molecular structure qualified as a puckered layer. In particular, the HC_2_O_4_^−^ ions are linked *via* O—H⋯O hydrogen bonds [2.533 (1) Å], leading to an infinite chain along [100]. In the title salt, the HC_2_O_4_^−^ ions inter­act in the same manner but through slightly longer O—H⋯O hydrogen bonds [2.564 (1) Å]. In addition, the oxalic acid mol­ecules that co-crystallize with Me_2_NH_2_^+^·HC_2_O_4_^−^ act both as donors and acceptors of hydrogen bonds through N—H⋯O and O—H⋯O bonds with the Me_2_NH_2_^+^ cation and HC_2_O_4_^−^ anion, respectively. Consequently, the degree of supra­molecularity is increased here, resulting in a two-dimensional architecture parallel to (010), which is reinforced by a C—H⋯O hydrogen bond (Table 1 ▸ and Figs. 2 ▸ and 3 ▸).

## Synthesis and crystallization   

Crystals of the title compound were obtained by mixing in 20 ml methanol (98% purity) Me_2_NH (0.30 g, 6.67 mmol), H_2_C_2_O_4_ (0.60 g, 6.67 mmol) and Sn(*n*-Bu)_3_Cl (4.39 g, 13.33 mmol). Another experimental method is the reaction between the (Me_2_NH_2_)_2_C_2_O_4_ salt (0.50 g, 2.77 mmol), previously synthesized from oxalic acid and di­methyl­amine, and Sn(CH_3_)_3_Cl (0.28 g, 1.39 mmol) in 15 ml of ethanol (98% purity). In both cases, the reaction mixture was stirred for *ca* 2 h at room temperature. Colourless crystals were obtained after one week by slow evaporation of the solvent.

## Refinement   

Crystal data, data collection and structure refinement details are summarized in Table 2 ▸. All the H atoms were placed in calculated positions and refined as riding: O—H = 0.84 Å, N—H = 0.91 Å, and C—H = 0.98 Å with *U*_iso_(H) = 1.5*U*_eq_(C,O) and 1.2*U*_eq_(N).

## Supplementary Material

Crystal structure: contains datablock(s) global, I. DOI: 10.1107/S2056989015005964/su5097sup1.cif

Structure factors: contains datablock(s) I. DOI: 10.1107/S2056989015005964/su5097Isup2.hkl

CCDC reference: 1055825

Additional supporting information:  crystallographic information; 3D view; checkCIF report

## Figures and Tables

**Table 1:** Hydrogen-bond geometry (, )

*D*H*A*	*D*H	H*A*	*D* *A*	*D*H*A*
O3H3O1^i^	0.84	1.73	2.564(1)	174
O5H5O2	0.84	1.73	2.565(1)	170
N1H1*A*O1^ii^	0.91	2.08	2.854(1)	143
N1H1*A*O4^ii^	0.91	2.23	2.964(1)	137
N1H1*B*O6	0.91	2.05	2.846(1)	146
C5H5*C*O4^iii^	0.98	2.41	3.346(1)	159

Symmetry codes: (i) 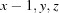; (ii) 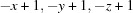; (iii) 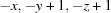.

**Table 2:** Experimental details

Crystal data	
Chemical formula	C_2_H_8_N^+^C_2_HO_4_ 0.5C_2_H_2_O_4_
*M*_r_	180.14
Crystal system, space group	Triclinic, *P* 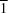
Temperature (K)	100
*a*, *b*, *c* ()	5.6519(3), 7.5809(4), 10.3100(6)
, , ()	75.467(2), 88.120(2), 69.487(2)
*V* (^3^)	399.76(4)
*Z*	2
Radiation type	Mo *K*
(mm^1^)	0.14
Crystal size (mm)	0.5 0.3 0.1

Data collection	
Diffractometer	Bruker D8 Venture triumph Mo
Absorption correction	Multi-scan (*SADABS*; Bruker, 2014 ▸)
*T*_min_, *T*_max_	0.693, 0.746
No. of measured, independent and observed [*I* 2(*I*)] reflections	10413, 1840, 1655
*R*_int_	0.023
(sin /)_max_ (^1^)	0.651

Refinement	
*R*[*F* ^2^ > 2(*F* ^2^)], *wR*(*F* ^2^), *S*	0.028, 0.075, 1.07
No. of reflections	1840
No. of parameters	113
H-atom treatment	H-atom parameters not refined
_max_, _min_ (e ^3^)	0.38, 0.26

Computer programs: *APEX2* and *SAINT* (Bruker, 2014 ▸), *SUPERFLIP* (Palatinus Chapuis, 2007 ▸), *SHELXL2014* (Sheldrick, 2015 ▸), *OLEX2* (Dolomanov *et al.*, 2009 ▸) and *Mercury* (Macrae *et al.*, 2008 ▸.

## supplementary crystallographic information

### Crystal data

**Table:**

C_2_H_8_N^+^·C_2_HO_4_^−^·0.5C_2_H_2_O_4_	*Z* = 2
*M**_r_* = 180.14	*F*(000) = 190.1598
Triclinic, *P*1	*D*_x_ = 1.497 Mg m^−^^3^
Hall symbol: -P 1	Mo *K*α radiation, λ = 0.71073 Å
*a* = 5.6519 (3) Å	Cell parameters from 7049 reflections
*b* = 7.5809 (4) Å	θ = 3.0–27.6°
*c* = 10.3100 (6) Å	µ = 0.14 mm^−^^1^
α = 75.467 (2)°	*T* = 100 K
β = 88.120 (2)°	Prism, colourless
γ = 69.487 (2)°	0.5 × 0.3 × 0.1 mm
*V* = 399.76 (4) Å^3^	

### Data collection

**Table:**

Bruker D8 Venture triumph Mo diffractometer	1840 independent reflections
Radiation source: X-ray tube, Siemens KFF Mo 2K-90C	1655 reflections with *I*≥ 2σ(*I*)
TRIUMPH curved crystal monochromator	*R*_int_ = 0.023
φ and ω scans	θ_max_ = 27.6°, θ_min_ = 3.0°
Absorption correction: multi-scan (*SADABS*; Bruker, 2014)	*h* = −7→7
*T*_min_ = 0.693, *T*_max_ = 0.746	*k* = −9→9
10413 measured reflections	*l* = −13→13

### Refinement

**Table:**

Refinement on *F*^2^	0 restraints
Least-squares matrix: full	Hydrogen site location: inferred from neighbouring sites
*R*[*F*^2^ > 2σ(*F*^2^)] = 0.028	H-atom parameters not refined
*wR*(*F*^2^) = 0.075	*w* = 1/[σ^2^(*F*_o_^2^) + (0.0375*P*)^2^ + 0.1325*P*] where *P* = (*F*_o_^2^ + 2*F*_c_^2^)/3
*S* = 1.07	(Δ/σ)_max_ = 0.001
1840 reflections	Δρ_max_ = 0.38 e Å^−^^3^
113 parameters	Δρ_min_ = −0.26 e Å^−^^3^

### Special details

**Table:**

Geometry. All e.s.d.'s (except the e.s.d. in the dihedral angle between two l.s. planes) are estimated using the full covariance matrix. The cell e.s.d.'s are taken into account individually in the estimation of e.s.d.'s in distances, angles and torsion angles; correlations between e.s.d.'s in cell parameters are only used when they are defined by crystal symmetry. An approximate (isotropic) treatment of cell e.s.d.'s is used for estimating e.s.d.'s involving l.s. planes.

### Fractional atomic coordinates and isotropic or equivalent isotropic displacement parameters (Å^2^)

**Table:**

	*x*	*y*	*z*	*U*_iso_*/*U*_eq_	
O1	0.75407 (12)	0.32103 (10)	0.47166 (7)	0.01407 (16)	
O2	0.57685 (12)	0.40754 (10)	0.26360 (7)	0.01329 (16)	
O3	0.14369 (12)	0.38767 (11)	0.36954 (7)	0.01625 (17)	
H3	0.0215	0.3632	0.4083	0.024*	
O4	0.36051 (14)	0.20987 (11)	0.56626 (7)	0.02010 (17)	
O5	0.29036 (13)	0.37192 (10)	0.09144 (7)	0.01471 (16)	
H5	0.3683	0.3944	0.1497	0.022*	
O6	0.01961 (13)	0.67729 (10)	0.07499 (7)	0.01568 (16)	
C1	0.57857 (16)	0.34999 (13)	0.38821 (9)	0.01061 (18)	
C2	0.34558 (17)	0.30728 (13)	0.45205 (9)	0.01216 (19)	
C3	0.08336 (17)	0.52128 (13)	0.04793 (9)	0.01171 (19)	
N1	0.22327 (15)	0.83866 (11)	0.24521 (8)	0.01223 (17)	
H1A	0.3034	0.7852	0.3284	0.015*	
H1B	0.1697	0.7490	0.2236	0.015*	
C4	0.40571 (19)	0.88277 (15)	0.14735 (10)	0.0182 (2)	
H4A	0.3224	0.9359	0.0569	0.027*	
H4B	0.5500	0.7630	0.1500	0.027*	
H4C	0.4653	0.9785	0.1705	0.027*	
C5	−0.00007 (19)	1.01362 (14)	0.24892 (11)	0.0177 (2)	
H5A	−0.0886	1.0711	0.1598	0.027*	
H5B	0.0557	1.1091	0.2751	0.027*	
H5C	−0.1149	0.9761	0.3143	0.027*	

### Atomic displacement parameters (Å^2^)

**Table:**

	*U*^11^	*U*^22^	*U*^33^	*U*^12^	*U*^13^	*U*^23^
O1	0.0100 (3)	0.0212 (4)	0.0119 (3)	−0.0072 (3)	−0.0008 (2)	−0.0031 (3)
O2	0.0113 (3)	0.0178 (3)	0.0107 (3)	−0.0060 (3)	−0.0004 (2)	−0.0024 (3)
O3	0.0089 (3)	0.0274 (4)	0.0131 (3)	−0.0087 (3)	0.0002 (3)	−0.0030 (3)
O4	0.0162 (4)	0.0286 (4)	0.0148 (3)	−0.0123 (3)	−0.0008 (3)	0.0022 (3)
O5	0.0134 (3)	0.0149 (3)	0.0150 (3)	−0.0021 (3)	−0.0044 (3)	−0.0059 (3)
O6	0.0178 (3)	0.0130 (3)	0.0166 (3)	−0.0046 (3)	−0.0040 (3)	−0.0051 (3)
C1	0.0087 (4)	0.0105 (4)	0.0127 (4)	−0.0028 (3)	0.0003 (3)	−0.0039 (3)
C2	0.0103 (4)	0.0154 (4)	0.0126 (4)	−0.0057 (3)	0.0004 (3)	−0.0050 (3)
C3	0.0127 (4)	0.0138 (4)	0.0091 (4)	−0.0059 (3)	−0.0003 (3)	−0.0018 (3)
N1	0.0145 (4)	0.0118 (4)	0.0107 (4)	−0.0050 (3)	−0.0005 (3)	−0.0028 (3)
C4	0.0166 (5)	0.0202 (5)	0.0186 (5)	−0.0074 (4)	0.0050 (4)	−0.0055 (4)
C5	0.0152 (5)	0.0148 (4)	0.0211 (5)	−0.0037 (4)	0.0033 (4)	−0.0034 (4)

### Geometric parameters (Å, º)

**Table:**

O1—C1	1.2573 (11)	N1—H1A	0.9100
O2—C1	1.2480 (11)	N1—H1B	0.9100
O3—H3	0.8400	N1—C4	1.4822 (12)
O3—C2	1.3089 (11)	N1—C5	1.4842 (12)
O4—C2	1.2105 (12)	C4—H4A	0.9800
O5—H5	0.8400	C4—H4B	0.9800
O5—C3	1.3051 (11)	C4—H4C	0.9800
O6—C3	1.2111 (12)	C5—H5A	0.9800
C1—C2	1.5515 (13)	C5—H5B	0.9800
C3—C3^i^	1.5501 (17)	C5—H5C	0.9800
			
C2—O3—H3	109.5	C5—N1—H1A	109.0
C3—O5—H5	109.5	C5—N1—H1B	109.0
O1—C1—C2	114.27 (8)	N1—C4—H4A	109.5
O2—C1—O1	126.60 (8)	N1—C4—H4B	109.5
O2—C1—C2	119.13 (8)	N1—C4—H4C	109.5
O3—C2—C1	112.45 (8)	H4A—C4—H4B	109.5
O4—C2—O3	126.54 (9)	H4A—C4—H4C	109.5
O4—C2—C1	121.01 (8)	H4B—C4—H4C	109.5
O5—C3—C3^i^	111.64 (10)	N1—C5—H5A	109.5
O6—C3—O5	126.87 (8)	N1—C5—H5B	109.5
O6—C3—C3^i^	121.48 (10)	N1—C5—H5C	109.5
H1A—N1—H1B	107.8	H5A—C5—H5B	109.5
C4—N1—H1A	109.0	H5A—C5—H5C	109.5
C4—N1—H1B	109.0	H5B—C5—H5C	109.5
C4—N1—C5	112.87 (8)		
			
O1—C1—C2—O3	162.31 (8)	O2—C1—C2—O3	−17.79 (12)
O1—C1—C2—O4	−17.45 (13)	O2—C1—C2—O4	162.45 (9)

Symmetry code: (i) −*x*, −*y*+1, −*z*.

### Hydrogen-bond geometry (Å, º)

**Table:**

*D*—H···*A*	*D*—H	H···*A*	*D*···*A*	*D*—H···*A*
O3—H3···O1^ii^	0.84	1.73	2.564 (1)	174
O5—H5···O2	0.84	1.73	2.565 (1)	170
N1—H1*A*···O1^iii^	0.91	2.08	2.854 (1)	143
N1—H1*A*···O4^iii^	0.91	2.23	2.964 (1)	137
N1—H1*B*···O6	0.91	2.05	2.846 (1)	146
C5—H5*C*···O4^iv^	0.98	2.41	3.346 (1)	159

Symmetry codes: (ii) *x*−1, *y*, *z*; (iii) −*x*+1, −*y*+1, −*z*+1; (iv) −*x*, −*y*+1, −*z*+1.
